# ﻿A new species of *Gaillardiellus* Guinot, 1976 (Crustacea, Brachyura, Xanthidae) from the coral reefs of the South China Sea

**DOI:** 10.3897/zookeys.1234.144026

**Published:** 2025-04-08

**Authors:** Yuan Ziming, Jiang Wei, Sha Zhongli

**Affiliations:** 1 Department of Marine Organism Taxonomy and Phylogeny, Institute of Oceanology, Chinese Academy of Sciences, Qingdao 266071, China; 2 Laboratory for Marine Biology and Biotechnology, Qingdao Marine Science and Technology Center, Qingdao 266237, China; 3 Shandong Province Key Laboratory of Experimental Marine Biology, Institute of Oceanology, Chinese Academy of Sciences, Qingdao 266071, China; 4 University of Chinese Academy of Sciences, Beijing 100049, China

**Keywords:** Actaeinae, COI, *Gaillardiellus*, identification key, morphology, new species, rock crabs, taxonomy

## Abstract

A new xanthid species of *Gaillardiellus* Guinot, 1976, is described from the coral reefs of the Xisha and Nansha Islands in the South China Sea. The new species, *Gaillardiellusmagiruber***sp. nov.**, closely resembles *G.rueppellii* (Krauss, 1843) but can be distinguished mainly by its closer proximity of the outer orbital angle and anterolateral margin, which lacks an accessory lobe, a broader and non-protruding front, and notable differences in live coloration and size. Molecular analysis of mitochondrial cytochrome *c* oxidase I (COI) sequences further corroborates the validity of this new species. An updated identification key for *Gaillardiellus* is provided.

## ﻿Introduction

The xanthid genus *Gaillardiellus* was first described by Guinot (1976), who included four species (including *G.rueppellii* (Krauss, 1843) as the type species), which were previously known under *Actaea* De Haan, 1833. The main distinguishing characteristics of *Gaillardiellus* include a carapace divided into distinct regions, an anterolateral margin subdivided into three or four lobes, pereiopods that lack nodules except for those on the carpus of the chelipeds, and distinctly sinuous margins of male pleonites 3–5, which fit closely with the corresponding parts of the thoracic sternum (Guinot 1976).

Currently, six species of *Gaillardiellus* are known worldwide: *G.rueppellii*, *G.alphonsi* (Nobili, 1905), *G.orientalis* (Odhner, 1925), *G.superciliaris* (Odhner, 1925), *G.bathus* Davie, 1997, and *G.holthuisi* Takeda & Komatsu, 2010 (Ng et al. 2008; Takeda and Komatsu 2010). These species inhabit the Indo-West Pacific region, ranging from intertidal zone to bathyal zone exceeding 300 m depth (Davie 1997).

In a recent coral reef biodiversity survey, a new species of *Gaillardiellus*, *G.magiruber* sp. nov., was discovered from the Xisha and Nansha Islands in the South China Sea. Here, we provide a detailed description of this species, supported by mitochondrial cytochrome *c* oxidase I (COI) sequence data to confirm its validity. Additionally, we present an updated identification key for the genus.

## ﻿Material and methods

### ﻿Morphological analyses

Material was collected by diving or remotely operated vehicle (ROV) from coral reefs in the South China Sea and preserved in 70% ethanol. The specimens were deposited at the Marine Biological Museum, Chinese Academy of Sciences (MBMCAS) in Qingdao, China, and were assigned catalogue numbers with the MBM prefix. A specimen from the Institute of Zoology, Chinese Academy of Sciences, was also examined and assigned catalogue numbers with the IOZ prefix. Morphological characteristics were observed using a ZEISS SteREO Discovery V20 stereoscopic microscope. Photographs were taken with a Canon EOS 6D camera equipped with a Canon MP-E 65 mm lens or a ZEISS Axiocam 506 microscope camera.

The terminology used primarily follows Serène (1984) and Davie et al. (2015). The following abbreviations are used in the text: CW = maximum carapace width; CL = median carapace length; G1 = first gonopod of male; G2 = second gonopod of male.

### ﻿Phylogenetic analyses

Genomic DNAs were extracted from muscle tissue by OMEGA EZNA Tissue DNA Kit. Mitochondrial cytochrome *c* oxidase I (COI, 658 bp) sequences were obtained for molecular phylogenetic analyses and amplified by polymerase chain reaction (PCR) with the primers jgLCO1490 and jgHCO2198 (Geller et al. 2013) or Pano-F and Pano-R (Thoma et al. 2014). The PCR 25 μl volumes contained: 1 μl (3–200 ng) of genomic DNA template, 1 μl (10 pM) of each primer, 12 μl of 2×PCR Mix (Dongsheng Biotech, Guangzhou, China), and 10 μl ultrapure water. Reactions were carried out with initial denaturation at 94 °C for 3 min, 35 cycles for denaturation at 94 °C for 30 s, annealing at 48 °C for 45 s, extension at 72 °C for 45 s, and final extension at 72 °C for 10 min.

Sixteen sequences representing 10 species were used for the analysis (Table 1). The sequences with the ZRC prefix originated from specimens from the Zoological Reference Collection of the Lee Kong Chian Natural History Museum (LKCNHM), National University of Singapore, Singapore. The nucleotide sequences were aligned using Muscle default settings in MEGA v. 6.06 (Tamura et al. 2013). The phylogenetic trees were reconstructed using maximum likelihood (ML) and Bayesian Inference (BI) algorithms. The best-fitting model was selected using jModeltest v. 0.1.1 under the Akaike information criterion (AIC) (Posoda 2008). The BI analyses were performed using MrBayes v. 3.2.7 (Huelsenbeck and Ronquist 2001), utilizing a Markov Chain Monte Carlo (MCMC) algorithm. Two independent runs were conducted, each comprising four chains over 1,000,000 generations, with tree sampling every 500 generations, resulting in 2000 sampled trees. The first 500 trees were discarded as burn-in, and posterior probabilities were calculated based on the remaining trees. The ML analyses were conducted online using W-IQ-TREE (http://iqtree.cibiv.univie.ac.at/) (Trifinopoulos et al. 2016), with clade support evaluated via 10,000 ML bootstrap replications.

**Table 1.** Species and sequences used in the phylogenetic analysis with GenBank accession numbers.

Catalogue number	Voucher ID	GenBank accession number	Species	Location	Reference
MBM288134	NS-MJ-2022-1859	PQ195874	*Gaillardiellusmagiruber* sp. nov.	China: Mischief Reef, Nansha Islands	present study
MBM288135	XS-QL-2022-1030	PQ195876	*Gaillardiellusmagiruber* sp. nov.	China: Qilianyu Islands, Xisha Islands	present study
MBM288142	G01	PQ195869	*Gaillardiellusorientalis* (Odhner, 1925)	China: Qingdao, Shandong	present study
ZRC 2000.1196	ZRC 2000.1196	HM750987	*Gaillardiellusorientalis* (Odhner, 1925)	Singapore: Palau Seringat	Lai et al. 2011
MBM283642	MBM283642	PQ195870	*Gaillardiellusrueppellii* (Krauss, 1843)	China: Weizhou Island, Guangxi	present study
ZRC 2010.0162	ZRC 2010.0162	HM750988	*Gaillardiellusrueppellii* (Krauss, 1843)	Philippines: Bohol Island	Lai et al. 2011
N.A.	PH	OP759455	*Gaillardiellusrueppellii* (Krauss, 1843)	South Korea: Jodo Island, Yeongdo-gu, Busan	unpublished
MBM288143	2404188881	PQ195868	*Gaillardiellussuperciliaris* (Odhner, 1925)	China: Zhongbei Shoal, Zhongsha Islands	present study
N.A.	UF:Invertebrate Zoology:45484-Arthropoda	MW277787	*Gaillardiellussuperciliaris* (Odhner, 1925)	USA:Patch Reef, Kaneohe Bay, Oahu, Hawaii	unpublished
MBM288144	XS-QL-2022-1108	PQ195877	*Paractaeatumulosa* (Odhner, 1925)	China: Qilianyu Islands, Xisha Islands	present study
MBM288145	G03	PQ195871	Paractaeacf.excentrica Guinot, 1969	China: Tree Island, Xisha Islands	present study
MBM288146	JN01	PQ195872	*Paractaearetusa* (Nobili, 1906)	China: Fiery Cross Reef, Nansha Islands	present study
MBM288147	JN02	PQ195873	*Paractaearetusa* (Nobili, 1906)	China: Tree Island, Xisha Islands	present study
MBM288148	NI01	PQ195875	*Paractaeaplumosa* Guinot in Sakai, 1976	China: Phoenix Island, Hainan	present study
MBM288149	ZS-MB-2022-1022	OP718532	*Trapezialutea* Castro, 1997	China: Walker Shoal, Zhongsha Islands	Yuan et al. 2023
MBM288150	XS-YL-2022-1012	OP718531	*Trapeziadigitalis* Latreille, 1828	China: Yongle Islands, Xisha Islands	Yuan et al. 2023

Multiple species delimitation methods were employed to evaluate the hypothesis that the specimens represent a distinct species. COI data were analyzed using the Automated Barcode Gap Discovery (ABGD) method via the ABGD web-based platform (https://bioinfo.mnhn.fr/abi/public/abgd/abgdweb.html) as described by Puillandre et al. (2012). The analysis was performed using the Kimura 2-parameter substitution model (TS/TV = 2.0), with the prior for maximum intraspecific divergence set between 0.001 and 0.1, over 10 recursive steps, and a relative gap width (X) of 1.0. Additionally, the Bayesian implementation of the Poisson Tree Processes (bPTP) species delimitation model was applied following Zhang et al. (2013). This analysis was conducted on the web server of the Heidelberg Institute for Theoretical Studies, Germany (http://species.h-its.org/), utilizing BI phylogenetic trees as input data.

## ﻿Taxonomic account

**Family Xanthidae MacLeay, 1838**

**Subfamily Actaeinae Alcock, 1898**

**Genus *Gaillardiellus* Guinot, 1976**

### ﻿ Gaillardiellus magiruber sp. nov.

Taxon classificationAnimaliaBrachyuraXanthidae

578CCEE8-4572-5897-B4A4-2FE8550FC5F9

https://zoobank.org/0D5CECEE-4112-4E55-8537-620637B3BE52

**Figure 1.**
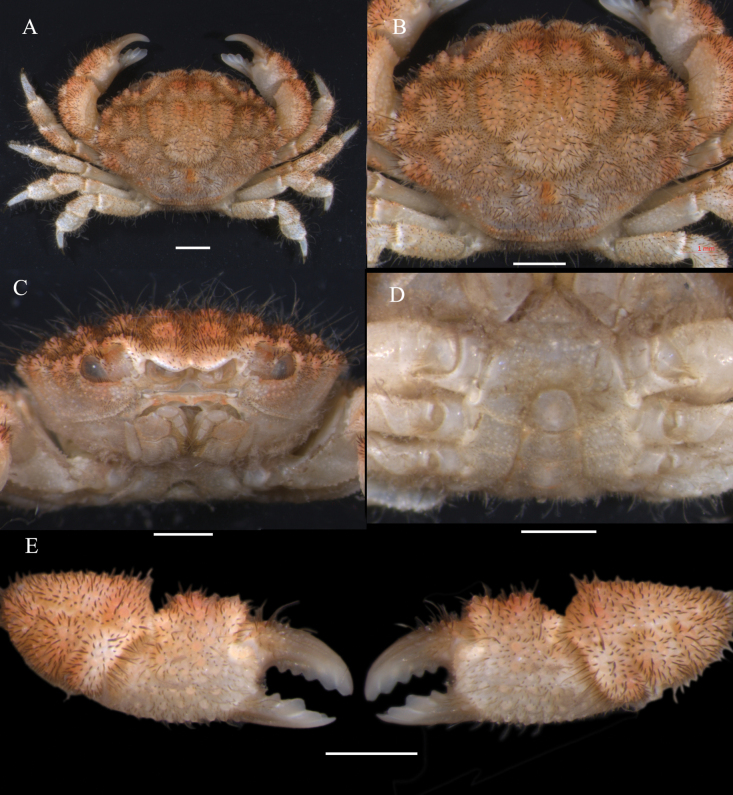
*Gaillardiellusmagiruber* sp. nov., male holotype (5.9 × 4.4 mm) (MBM288133) **A** overall dorsal view **B** dorsal view of cephalothorax **C** frontal view of cephalothorax **D** thoracic sternites, pleon and telson **E** outer view of chelipeds. Scale bar: 1 mm.

**Figure 2.**
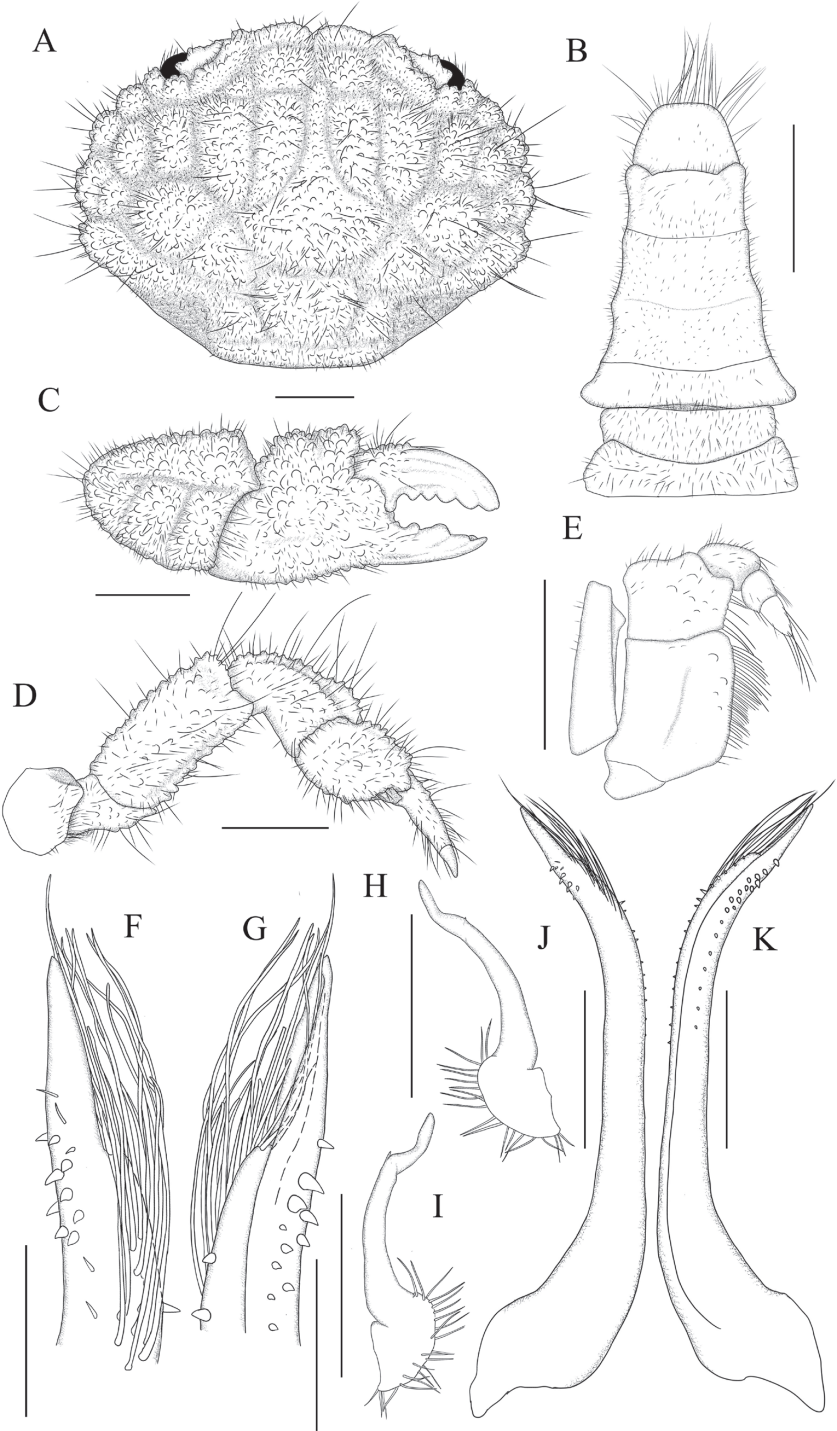
*Gaillardiellusmagiruber* sp. nov., male holotype (5.9 × 4.4 mm) (MBM288133) **A** dorsal view of cephalothorax **B** pleon and telson **C** outer view of right cheliped **D** right pereiopod 5 **E** right third maxilliped **F** dorsal view of left G1 distal part **G** ventral view of left G1 distal part **H** dorsal view of left G2 **I** ventral view of left G2 **J** dorsal view of left G1 **K** ventral view of left G1. Scale bars: 1 mm (**A–E**); 0.2 mm (**F**, **G**); 0.5 mm (**H–K**).

**Figure 3.**
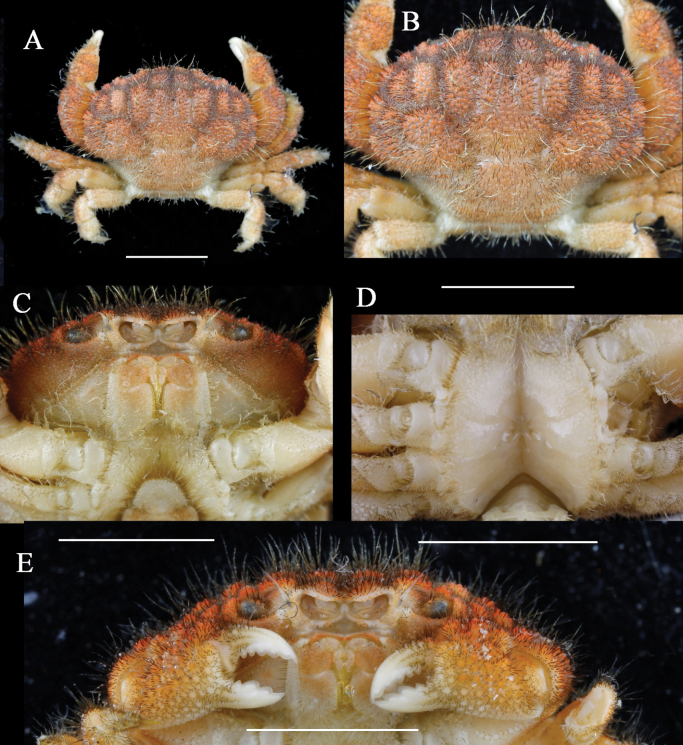
*Gaillardiellusmagiruber* sp. nov., female paratype (9.8 × 7.1 mm) (MBM288134) **A** overall dorsal view **B** dorsal view of cephalothorax **C** subhepatic and pterygostomial regions **D** thoracic sternum showing vulvae **E** outer view of chelipeds. Scale bar: 5 mm.

**Figure 4.**
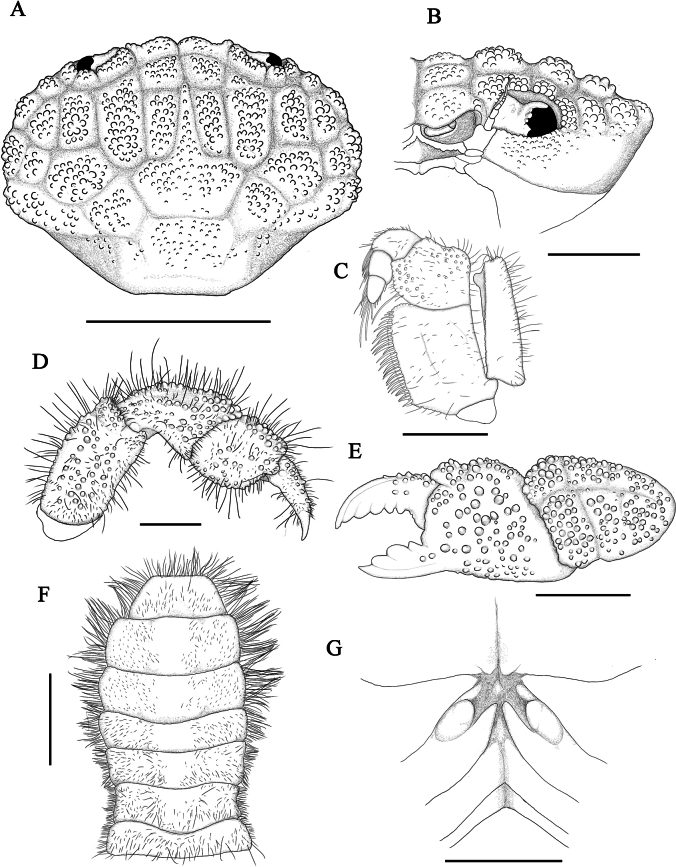
*Gaillardiellusmagiruber* sp. nov., female paratype (9.8 × 7.1 mm) (MBM288134) **A** dorsal view of cephalothorax, setae removed **B** frontal view of left half of cephalothorax, setae removed **C** left third maxilliped **D** right pereiopod 5 **E** outer view of left cheliped, setae removed **F** pleon and telson **G** vulvae. Scale bars: 5 mm (**A**); 2 mm (**B**, **E**, **F**); 1 mm (**C**, **D**, **G**).

**Figure 5.**
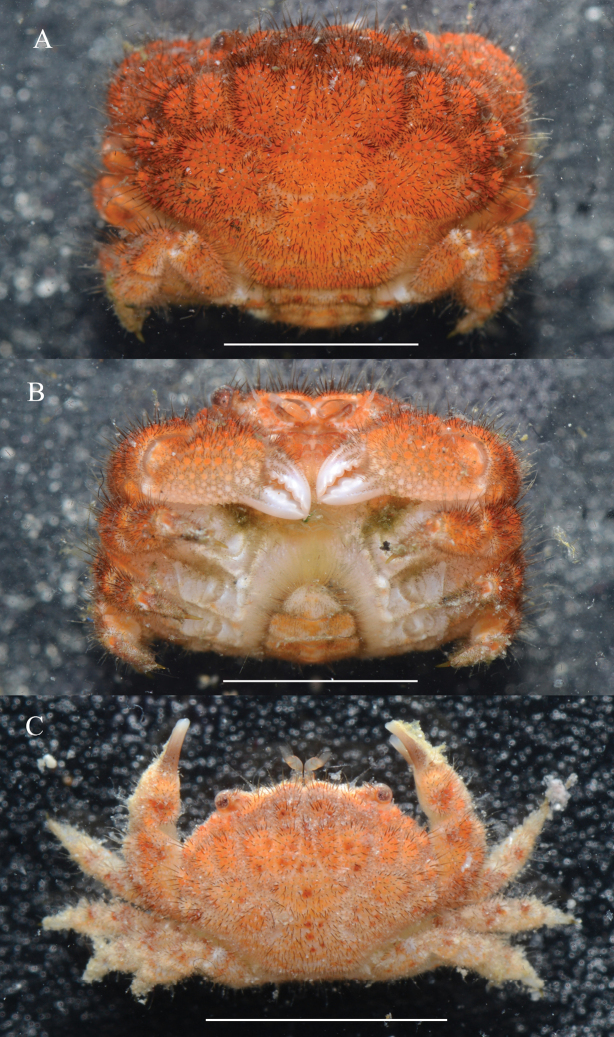
*Gaillardiellusmagiruber* sp. nov., live coloration **A**, **B** female paratype (9.8 × 7.1 mm) (MBM288134) **C** female paratype (5.7 × 4.2 mm) (MBM288135). Scale bar: 5 mm.

Figs 1 , 2 , 3 , 4 , 5 , Suppl. material 1

#### Material examined.

***Holotype***: China • ♂, 5.9 × 4.4 mm; Xisha Islands, Tree Island; depth 58 m; 2 Jul. 1977; Dong Dong leg.; MBM288133. ***Paratypes***: China • 1 ♀, 9.8 × 7.1 mm; Nansha Islands, Mischief reef; 9°54'N, 115°34'E; depth 1 m; 11 May 2022; Yuan Ziming, Sun Yuli, Ma Shaobo leg.; MBM288134 • 1 ♀, 9.5 × 6.4 mm; Nansha Islands, Banyue Reef; 29 Sep. 1994; MBM164284 • 1 ♀, 5.7 × 4.2 mm; Xisha Islands, Qilianyu Islands; 16°58'N, 112°16'E; depth 10 m; 19 May 2022; Yuan Ziming, Sun Yuli, Ma Shaobo leg.; MBM288135 • 1 juvenile ♂, 4.5 × 3.5 mm; Xisha Islands, North Reef; depth 30 m; 1 Sep. 2024; Dong Dong leg.; MBM288136 • 1 juvenile ♂, 4.2 × 3.3 mm; Xisha Islands, Huaguang Reef; depth 31 m; 26 Aug. 2024; Dong Dong leg.; MBM288137 • 1 juvenile ♀, 4.5 × 3.4 mm; Xisha Islands, Yagong Island; depth 33 m; 19 Aug. 2024; Dong Dong leg.; MBM288138 • 1 juvenile ♀, 4.1 × 3.0 mm; Xisha Islands, Yagong Island; depth 31 m; 20 Aug. 2024; Dong Dong leg.; MBM288139.

#### Comparative material.

**Figure 6.**
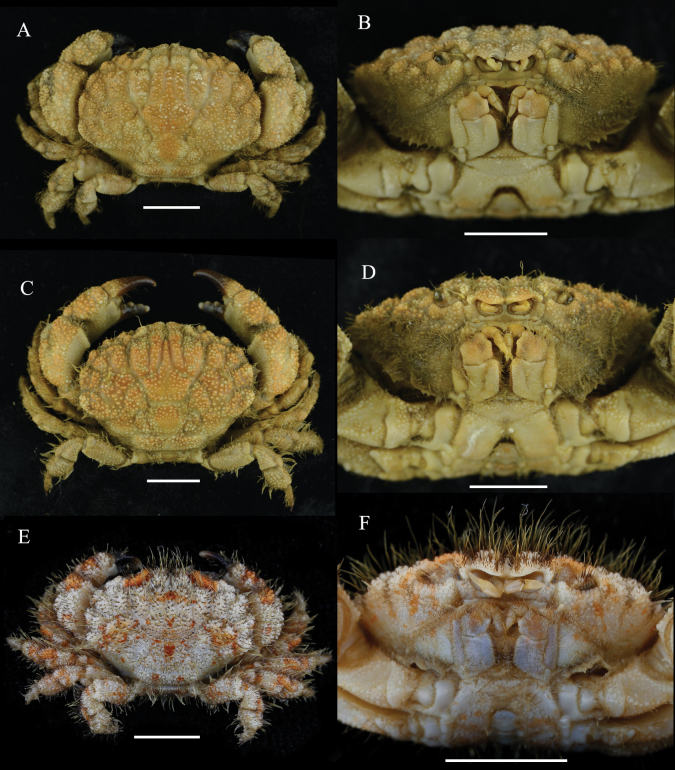
Comparative material of *Gaillardiellus* species: *Gaillardiellusrueppellii* (Krauss, 1843), male (38.6 × 28.2 mm) (MBM283642) (**A**, **B**) *Gaillardiellusorientalis* (Odhner, 1925), male (34.9 × 25.8 mm) (MBM288142) (**C**, **D**) *Gaillardiellussuperciliaris* (Odhner, 1925), male (11.6 × 7.9 mm) (MBM288143) (**E**, **F**) **A**, **C**, **E** overall dorsal view **B**, **D**, **F** frontal view of cephalothorax. Scale bars: 10 mm (**A–D**); 5 mm (**E**, **F**).

**Figure 7.**
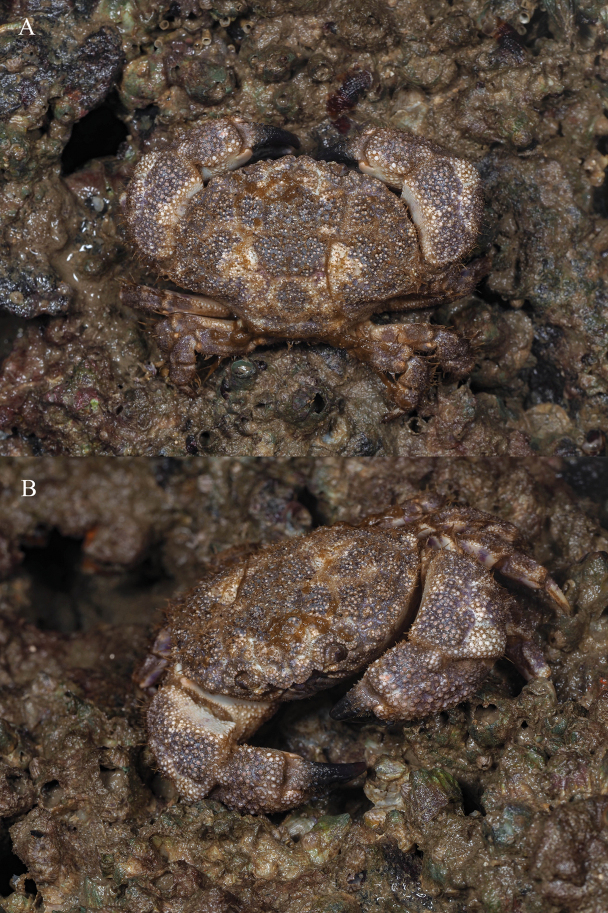
*Gaillardiellusrueppellii* (Krauss, 1843), live coloration, photo by Zhang Xu.

***Gaillardiellusrueppellii* (Krauss, 1843)** (Figs 6A, B, 7) China • 1 ♂; Xisha Islands, Money Island; 17 Mar. 1977; IOZ31604-01-4 • 1 ♂; Hainan, Dachan Island; 25 Mar. 2018; MBM288140 • 1 ♂; Hainan, Lingao Bay; depth 15–30 m; 20 Aug. 2018; Pan Yunhao leg.; MBM288141 • 1 ♂; Guangxi, Weizhou Island; 19 Nov. 2018; MBM283642. CW 25.3–45.6 mm, CL 19.4–34.4 mm. ***Gaillardiellusorientalis* (Odhner, 1925)** (Fig. 6C, D) China • 1 ♂, 34.9 × 25.8 mm; Shandong, Qingdao; 22 Jul. 2015; Yang Bin leg.; MBM288142. ***Gaillardiellussuperciliaris* (Odhner, 1925)** (Fig. 6E, F) China • 1 ♂, 11.6 × 7.9 mm; Zhongsha Islands, Zhongbei Shoal; 16°5'N, 114°25'E; 6 May 2024; Yuan Ziming leg.; MBM288143.

#### Diagnosis.

Carapace (Figs 1A, B, 2A, 3A, B, 4A, B) transversely oval, regions clearly defined, short setae present within grooves and between granules, long setae scattered between granules; front not protruding, slightly curved downwards, divided into 2 lobes by broad V-shaped notch; anterolateral margin divided into 4 granular lobes, first lobe small, slightly larger than outer orbital angle, adjacent to latter; posterolateral margin shorter than anterolateral margin, distinctly concave. Thoracic sternum (Figs 1D, 3D) with low granules, sternites 1 to 4 covered with soft setae. Male pleonite 6 (Figs 1D, 2B) with expanded lateral distal angles, wider than long; telson wider than long, terminal end blunt. G1 (Fig. 2F, G, J, K) curved outwards, distal third with small spines, long setae near distal end, terminal lobe slender. Orange-red to vibrant bright red in life (Fig. 5).

#### Description of male holotype.

Carapace (Figs 1A, B, 2A) transversely oval, CW about 1.3 times CL, dorsal surface slightly elevated, regions clearly defined, covered with granules, grooves wide and deep; short setae present within grooves and between granules, long setae scattered between granules; regions 1–3M distinct, 2M completely divided into 2 lobes, 3M intact, 4M indistinct; regions 2–6L distinct, 1P distinct, 2P indistinct; front about 0.3 times CW, not protruding, slightly curved downwards, divided into 2 lobes by broad V-shaped notch, inner lobes rounded and more prominent, outer lobes smaller and flatter, separated from inner orbital angle by notch; dorsal orbital margin with 2 sutures; eyestalks with setae and granules near cornea. Outer orbital angle not fused with anterolateral margin; anterolateral margin divided into 4 granular lobes, first lobe small, slightly larger than outer orbital angle, adjacent to latter, second and third lobes broader, fourth lobe smaller than third; posterolateral margin shorter than anterolateral margin, distinctly concave; subhepatic region with granules and short setae; pterygostomial region smooth with soft setae.

Antennule (Fig. 1C) folding transversely, antennular fossa subrectangular; basal segment of antenna subrectangular, filling orbital hiatus, antennal flagellum fitting into orbital hiatus. Epistome (Fig. 1C) central region with a strong median projection on posterior margin. Third maxilliped merus subquadrate, with low granules, anterior margin slightly indented, outer distal angle slightly expanded; ischium subrectangular, with submedian groove.

Chelipeds (Figs 1E, 2C) symmetrical, merus margins with low granules and soft setae; carpus robust, densely covered with granules and long setae, dorsolateral surface with grooves; palm densely covered with granules and setae on dorsal and lateral surfaces, ventral and medial surfaces smoother with low granules, dorsal surface with 2 granular tubercles; gap present when fingers closed; basal part of movable finger with granules, dorsal surface with 2 grooves, 3–5 rounded teeth between fingers, tips slightly concave.

Ambulatory legs (Figs 1A, 2D) densely covered with setae and granules; merus concave near terminal end of dorsal margin; carpus with groove near anterior margin, slightly swollen near terminal end; propodus nearly rhomboid; dactylus almost as long as propodus, terminal end chitinous, long, and sharp, with underdeveloped dactylo-propodal lock.

Thoracic sternum (Fig. 1D) with low granules, sternites 1 to 4 covered with soft setae; sternites 1 and 2 fused, suture between sternites 2 and 3 straight, suture between sternites 3 and 4 visible at margins, extending as shallow groove towards center, slightly curving backwards, with sternite 4 partially covered by telson, central groove beneath telson; tubercle of sterno-pleonal lock (press-button mechanism) located on anterior margin of sternite 5.

Pleon (Figs 1D, 2B) relatively short, pleonites 3 to 5 fused, fusion lines visible, margins concave and sinuous, fitting closely to corresponding part of thoracic sternum; pleonite 6 with expanded lateral distal angles, wider than long; telson (Figs 1D, 2B) wider than long, terminal end blunt, with long soft setae.

G1 (Fig. 2F, G, J, K) curved outward, distal third with small spines, long setae near distal end, terminal lobe slender. G2 (Fig. 2H, I) about one-third length of G1, curved outwards, terminal lobe longer and curved upwards.

#### Note on paratypes.

In the current paratypes, an adult female exhibits the largest body size (9.8 × 7.1 mm; MBM288134, Figs 3, 4, 5A, B). Compared to the male holotype, the female paratype possesses a broader and more expanded carapace and a more concave posterior margin (Figs 3A, B, 4A), which may reflect a higher level of maturity in this species. In smaller individuals and juveniles, the carapace is narrower.

The overall morphology of the female is similar to that of the male, with the following sexual dimorphic characteristics: female pleon broad, oval-shaped (Fig. 4F); telson terminal end blunt (Fig. 4F); and vulva located at the anterior margin of the sternite 6, with an oval-shaped cover (Figs 3D, 4G).

#### Colour in life.

In the current specimens, as body size increases, the coloration changes from a lighter orange-red with bright red spots (Fig. 5C) to a totally vibrant bright red (Fig. 5A). The cheliped fingers change from having a white distal half and a brown base (Fig. 5C) to being entirely white along their length (Fig. 5A).

#### Etymology.

The new species is named after the fiery Stand “Magician’s Red” from the manga “JoJo’s Bizarre Adventure”, wielded by the character Muhammad Avdol. This name alludes to the species’ changing flame-like red coloration.

#### Remarks.

*Gaillardiellusmagiruber* sp. nov. should be placed within *Gaillardiellus* based on the well-defined regions on the dorsal carapace, the morphology of the granules and setae, the presence of four granular rounded lobes on the anterolateral margin (Figs 1B, 2A), the absence of nodules on the ambulatory legs (Figs 1A, 2D), and the sinuous margins of male pleonites 3–5, along with the overall morphology of the thoracic sternum (Fig. 2B). The anterolateral margin of *G.magiruber* sp. nov. is completely separated from the outer orbital angle (Figs 1A, 2A, 3B, 4A), which distinguishes it clearly from *G.superciliaris* and *G.alphonsi* as their outer orbital angle is fused with the first anterolateral lobe (cf. Guinot 1976: pl. 16 figs 4, 5). Additionally, the well-developed long setae on the dorsal surface of the carapace and the evenly rounded teeth on the immovable finger of the cheliped further differentiate this new species from *G.holthuisi* and *G.bathus*. In contrast, *G.holthuisi* and *G.bathus* have only sparse long setae, and they also differ in the placement of a strong tooth: in *G.holthuisi*, it is near the tip of the immovable finger, while in *G.bathus*, it is in the middle part of the immovable finger (cf. Davie 1997: figs 1b, 15c; Takeda and Komatsu 2010: figs 1, 2B).

*Gaillardiellusmagiruber* sp. nov. is most similar to two closely related congeners, i.e., *G.rueppellii* and *G.orientalis*. Considering the scattered distribution of setae on the dorsal surface of the carapace, rather than the distinct tufted clusters in *G.orientalis* (cf. Guinot 1976: pl. 16, fig. 2), *G.magiruber* sp. nov. is especially similar to *G.rueppellii* and could be confused with it. *Gaillardiellusrueppellii* was first reported from Natal, South Africa and is widely known in the Indo-West Pacific region (Krauss 1843; Guinot 1976; Serène 1984). Guinot (1976) provided a detailed redescription of the type specimen, accompanied by refined photographs and illustrations (cf. Guinot 1976: figs 42A, 43A, 43a, 44B, pl. 16, fig. 1, 1a), enhancing the understanding of *G.rueppellii*. *Gaillardiellusmagiruber* sp. nov. can be distinguished from *G.rueppellii* by the following characteristics: the front being broader and non-protruding, about 0.3 times CW (Figs 1B, 2A) (vs. front narrower and protruding, about 0.2 times CW in *G.rueppellii*; Fig. 6A; cf. Guinot 1976: pl. 16, fig. 1); the first anterolateral lobe is almost adjacent to the outer orbital angle, with no additional lobe underneath (Figs 1B, C, 2A) (vs. a wider gap between the first anterolateral lobe and outer orbital angle, with an accessory lobe underneath in *G.rueppellii*; Fig. 6B; cf. Guinot 1976: pl. 16, fig. 1); the male and female thoracic sternum with long soft setae on sternites 1–4 (Figs 1D, 3D) (vs. only sparse short setae in *G.rueppellii*; Fig. 6B); the male pleon is shorter, the pleonite 6 and telson being broader than long, and the telson with long soft setae on tip (Figs 1D, 2B) (vs. male pleon is longer, the pleonite 6 and telson are nearly equal in length and width, and the telson without long soft setae in *G.rueppellii*; cf. Guinot 1976: fig. 62A); the G1 is shorter and stouter (Fig. 2J, K) (vs. G1 slender in *G.rueppellii*; cf. Guinot 1976: fig. 63A). Additionally, *G.magiruber* sp. nov. has a more vibrant bright red live coloration (Fig. 5) (vs. duller coloration, appearing brownish in *G.rueppellii*; Fig. 7).

Furthermore, although the present specimens of *G.magiruber* sp. nov. include some juvenile individuals, considering the body size of the two female specimens displaying distinct maturity traits, such as the well-developed pleon and vulva, the new species is relatively smaller in size (CW less than 10 mm) compared to most congeners.

It is worth noting that *G.rueppellii* has two early synonyms: *Actaeapilosa* Stimpson, 1858, from Hong Kong, and *Aeglerugata* Adams & White, 1849 (not H. Milne Edwards, 1834), from the Philippine Islands. Unfortunately, the type specimen of *Aeglerugata* is no longer traceable (Dr Paul Clark, Natural History Museum, personal communication), and the type specimen of *Actaeapilosa* was likely lost in the infamous fire. The identities of these two specimens remain uncertain. However, based on the limited available illustrations (Stimpson 1907: pl. 5 fig. 6; Adams and White 1849: pl. 8, fig. 5), both species exhibit a prominently lobed frontal margin, suggesting closer affinity to *G.rueppellii* rather than *G.magiruber* sp. nov. Due to the similarities between the two species, *G.magiruber* sp. nov. may be mistakenly identified as a juvenile of *G.rueppellii*. Further extensive examination will help clarify the distribution ranges of both species.

**Figure 8.**
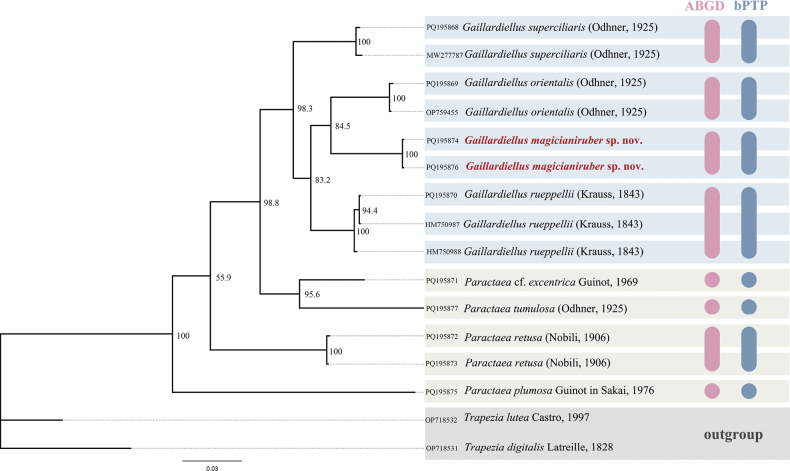
Bayesian inference (BI) phylogenetic tree based on COI showing the phylogenetic relationship between *Gaillardiellusmagiruber* sp. nov. and related species, with bootstrap replications (BS) labeled. The results of Automated Barcode Gap Discovery (ABGD) and Bayesian implementation of the Poisson Tree Processes (bPTP) species delimitation methods are shown on the right margin of the figure, each circle or capsule shape represents one species.

In the COI-based molecular analysis, the BI (Fig. 8) and ML (Suppl. material 2) trees exhibited similar topologies. *Gaillardiellusmagiruber* sp. nov. is most closely related to *G.orientalis*, followed by clustering with *G.rueppellii*. Species delimitation based on ABGD and bPTP further supports the validity of the new species.

In addition, *Paractaea* Guinot, 1969, is not monophyletic in the current study, with *P.tumulosa* (Odhner, 1925) and P.cf.excentrica (Guinot, 1969) forming a single clade. Serène (1984) previously suggested transferring *P.tumulosa* to *Paractaeopsis* Serène, 1984 (see Takeda and Komatsu 2018). Further research is needed to clarify the phylogenetic relationships among species within *Paractaea*.

#### Geographic distribution.

Xisha and Nansha Islands, South China Sea.

### ﻿Key to species of *Gaillardiellus* (adapted from Serène 1984)

**Table:** 

1	Carapace anterolateral margin with 3 distinct lobes behind outer orbital angle, anterior lobe fused with outer orbital angle; 3M faintly divided into 3 parts	***G.superciliaris* and *G.alphonsi* [possibly synonyms; see also Guinot 1976**]
–	Carapace anterolateral margin with 4 distinct lobes behind outer orbital angle, anterior lobe not fused with outer orbital angle; 3M intact	**2**
2	Cheliped immovable finger with strong tooth	**3**
–	Cheliped immovable finger without strong tooth	**4**
3	Cheliped with strong tooth near tip of immovable finger	***G.holthuisi***
–	Cheliped with strong tooth in middle part of immovable finger	***G.bathus***
4	Carapace dorsal surface with tufts of long, plumose setae	***G.orientalis***
–	Carapace dorsal surface with scattered sort and long setae	**5**
5	Carapace first anterolateral lobe widely separated from outer orbital angle, with accessory lobe located in-between	***G.rueppellii***
–	Carapace first anterolateral lobe almost adjacent to outer orbital angle, with no additional lobe underneath	***G.magiruber* sp. nov.**

## Supplementary Material

XML Treatment for Gaillardiellus magiruber

## ﻿Additional information

### Author ORCIDs

Yuan Ziming https://orcid.org/0009-0008-0009-2693

Jiang Wei https://orcid.org/0000-0003-4767-8196

Sha Zhongli https://orcid.org/0000-0002-2192-3758

### Data availability

All of the data that support the findings of this study are available in the main text or Supplementary Information.

## Supplementary materials

Supplementary material 1 *Gaillardiellusmagiruber* sp. nov. Data type jpg Explanation note Juvenile male paratype (4.5 × 3.5 mm) (MBM288136) (**A**–**C**); juvenile male paratype (4.2 × 3.3 mm) (MBM288137) (**D–F**). **A**, **D** dorsal view **B**, **E** ventral view **C**, **F** immature G1 and G2. Scale bars: 1 mm (**A**, **B**, **D**, **E**), 0.2 mm (**C**, **F**). https://binary.pensoft.net/file/1301421This dataset is made available under the Open Database License (http://opendatacommons.org/licenses/odbl/1.0/). The Open Database License (ODbL) is a license agreement intended to allow users to freely share, modify, and use this Dataset while maintaining this same freedom for others, provided that the original source and author(s) are credited.Yuan Ziming, Jiang Wei, Sha Zhongli

Supplementary material 2 Maximum likelihood (ML) phylogenetic tree based on COI showing the phylogenetic relationship between *Gaillardiellusmagiruber* sp. nov. and related species Data type jpg https://binary.pensoft.net/file/1301422This dataset is made available under the Open Database License (http://opendatacommons.org/licenses/odbl/1.0/). The Open Database License (ODbL) is a license agreement intended to allow users to freely share, modify, and use this Dataset while maintaining this same freedom for others, provided that the original source and author(s) are credited.Yuan Ziming, Jiang Wei, Sha Zhongli
