# Supplementary figures and images for: ﻿A new species of Gaillardiellus Guinot, 1976 (Crustacea, Brachyura, Xanthidae) from the coral reefs of the South China Sea
Source: Zookeys. 2025 Apr 8;1234:1–17. doi: 10.3897/zookeys.1234.144026 (PMC12000811; doi:10.3897/zookeys.1234.144026)

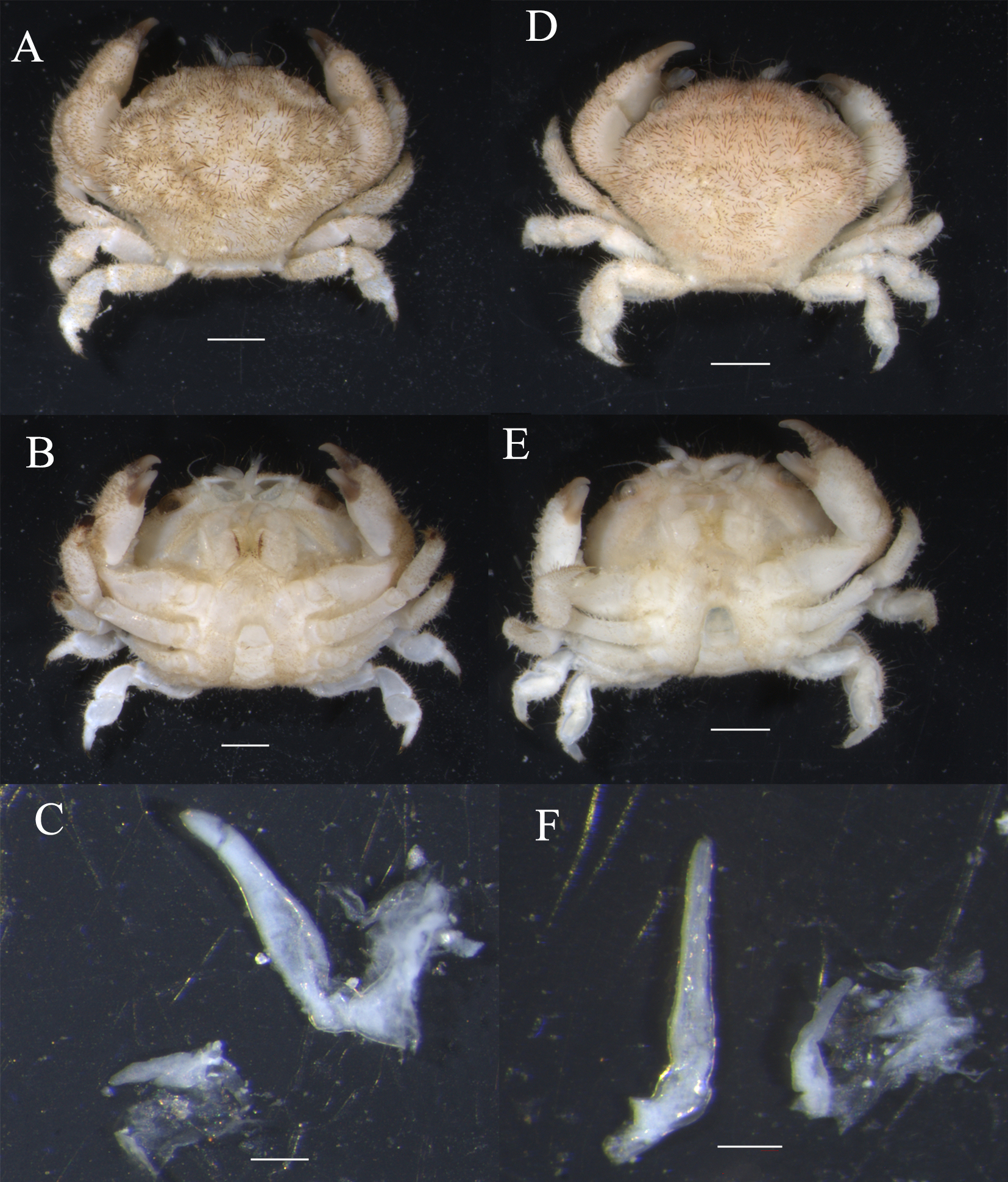

Supplement: Supplementary material 1 — Gaillardiellusmagiruber sp. nov. [file zookeys-1234-001_article-144026__-s001.jpg]

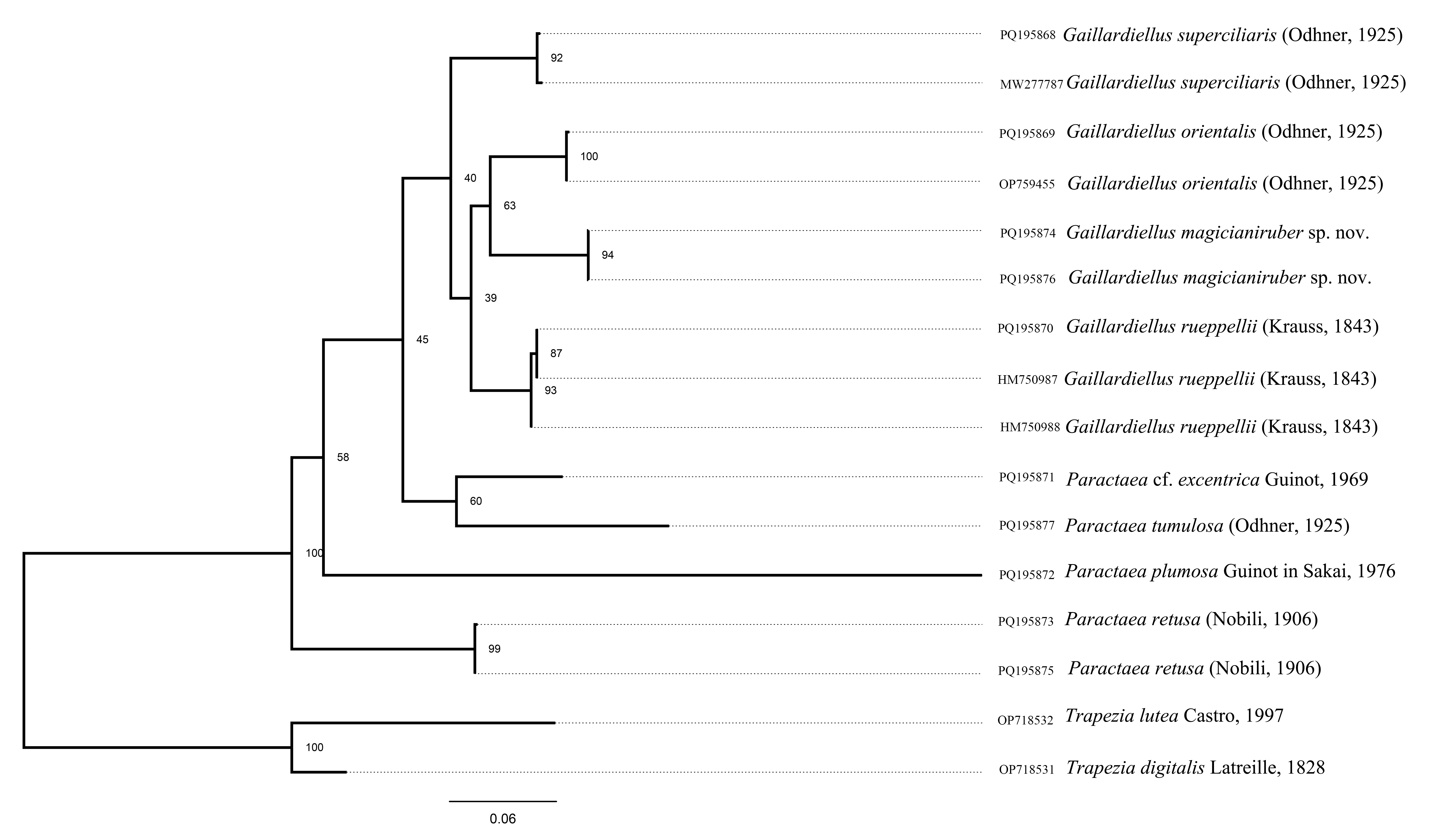

Supplement: Supplementary material 2 — Maximum likelihood (ML) phylogenetic tree based on COI showing the phylogenetic relationship between Gaillardiellusmagiruber sp. nov. and related species [file zookeys-1234-001_article-144026__-s002.jpg]
